# PDZ-binding kinase/T-LAK cell-originated protein kinase is a target of the fucoidan from brown alga *Fucus evanescens* in the prevention of EGF-induced neoplastic cell transformation and colon cancer growth

**DOI:** 10.18632/oncotarget.7708

**Published:** 2016-02-25

**Authors:** Olesia S. Vishchuk, Huimin Sun, Zhe Wang, Svetlana P. Ermakova, JuanJuan Xiao, Tao Lu, PeiPei Xue, Tatyana N. Zvyagintseva, Hua Xiong, Chen Shao, Wei Yan, Qiuhong Duan, Feng Zhu

**Affiliations:** ^1^ Department of Biochemistry and Molecular Biology, School of Basic Medicine, Huazhong University of Science and Technology, Wuhan, Hubei 430030, PR China; ^2^ G.B. Elyakov Pacific Institute of Bioorganic Chemistry, Far Eastern Branch of Russian Academy of Sciences, Laboratory of Enzyme Chemistry, 690022 Vladivostok, Russian Federation; ^3^ Department of Urology, Xijing Hospital, The Fourth Military Medical University, Xi'an, Shaanxi 710032, PR China; ^4^ Department of Pathology, Xijing Hospital, The Fourth Military Medical University, Xi'an, Shaanxi 710032, PR China; ^5^ State Key Laboratory of Cancer Biology & Xijing Hospital of Digestive Diseases, The Fourth Military Medical University, Xi'an, Shaanxi 710032, PR China

**Keywords:** fucoidan, Fucus evanescens, TOPK, cell transformation, colon carcinoma

## Abstract

The fucoidan with high anticancer activity was isolated from brown alga *Fucus evanescens*. The compound effectively prevented EGF-induced neoplastic cell transformation through inhibition of TOPK/ERK1/2/MSK 1 signaling axis. *In vitro* studies showed that the fucoidan attenuated mitogen-activated protein kinases downstream signaling in a colon cancer cells with different expression level of TOPK, resulting in growth inhibition. The fucoidan exerts its effects by directly interacting with TOPK kinase *in vitro* and *ex vivo* and inhibits its kinase activity. In xenograft animal model, oral administration of the fucoidan suppressed HCT 116 colon tumor growth. The phosphorylation of TOPK downstream signaling molecules in tumor tissues was also inhibited by the fucoidan. Taken together, our findings support the cancer preventive efficacy of the fucoidan through its targeting of TOPK for the prevention of neoplastic cell transformation and progression of colon carcinomas *in vitro* and *ex vivo*.

## INTRODUCTION

Cancer is considered to be a leading cause of death worldwide despite the intensive efforts and substantial advances that have occurred through focusing on improving treatments. Recently it was indicated that one-third of all cancer deaths are preventable and that diet is closely linked to cancer prevention. Chemoprevention may be defined as the use of non-toxic substances, including many food factors and natural compounds to interfere with the process of cancer development or carcinogenesis before invasion and metastasis can occur. On the basis of this idea, and numerous epidemiological findings, attention has centred on dietary natural compounds as an effective intervention in cancer development [1].

A great deal of interest has been developed in the nutraceutical and pharmaceutical industries to isolate natural biological active compounds from marine resources. Among marine organisms, brown algae belonging to *Saccharina*, *Fucus*, *Alaria*, *Sargassum*, *Undaria*, *Pelvetia* genera have traditionally formed part of the oriental diet, especially in Asian-pacific region, while the purified gelling and thickening ingredients are predominant as food products of algal origin in European countries and USA. Nowadays, algae have been marketed worldwide as constituents of dietary supplements due to their antimutagenic, anticoagulant, and antitumor properties as well as the high content of so-called dietary fiber [2]. Brown algae are known to produce a range of active components including unique secondary metabolites such as phlorotannins and polysaccharides, namely alginic acids, laminarans, and sulfated polysaccharides (fucoidans). The fucoidans are shown to be a topic of numerous studies as nontoxic compounds, possessing wide spectrum of biological activities [3, 4]. The fucoidans from different species of brown algae have been found to inhibit carcinogenesis in variety of cancer cells, including gastric adenocarcinoma [5], prostate cancer [6], melanoma [7], hepatocellular carcinoma [8], breast cancer [9], and colon cancer cells [10].

The key molecular mechanism of anticancer effect of the fucoidans is the induction of apotosis through caspase-dependent and caspase-independent pathways [11–14]. Moreover the fucoidans were shown to suppress tumor growth by inhibiting tumor-induced angiogenesis and metastasis [15]. The mechanism by which the fucoidans inhibited these processes has not been clearly elucidated. Probably, the fucoidans are responsible for the reduction of activities of Matrix Metalloproteinases (MMPs) and the decrease of Vascular Endothelial Growth Factor (VEGF) expression with subsequent inhibition of invasion and suppression of tubules formation in tumor cells [16–18].

While the development of research efforts involving structure of the fucoidans and their biological activities are advancing, the understanding of molecular mechanisms of their action is still incomplete. Moreover, a direct molecular target of the fucoidan from brown alga *Fucus evanescens* has not been identified *in vitro* or *ex vivo*.

The mitogen-activated protein kinase kinase (MAPKK) signaling pathway is a major component of the RAS/RAF/MEK/ERK signaling axis which is activated by a multitude of extracellular stimuli, including a variety of tumor promoters; and they participate in the regulation of a host of cellular functions such as transformation, proliferation and growth, cell movement, differentiation, and death [19].

The Lymphokine-activated killer T-cell-originated protein kinase (TOPK) is a serine-threonine kinase, a member of MAPKK family. TOPK was confirmed to highly express in many cancers such as lymphoma, leukemia, melanoma, colorectal, breast, lung cancer, and cholangiocarcinoma [20–22]. In addition, TOPK may contribute to oncogenic cellular functions including tumor development, cancer growth, and antiapoptotic effects [23–25]. On the basis of the above the TOPK kinase is certain to be a potential target for development of anticancer agents.

The aim of the present study was to elucidate molecular mechanism of chemopreventive effect of the fucoidan from brown alga *Fucus evanescens* and identify its direct molecular target.

## RESULTS

### The fucoidan inhibits EGF-induced neoplastic transformation of JB6 Cl41 cells through TOPK/ERK1/2/MSK 1 pathway

The carcinogenesis is multistage process, including initiation, promotion, and progression [26]. One of the perspective approaches for cancer therapy is search and development of nontoxic compounds, which are effective in preventing of cancer initiation.

The promotion-sensitive mouse epidermal cells JB6 Cl41 are known to respond irreversibly to tumor promoters such as epidermal growth factor (EGF) with induction of anchorage-independent growth in soft agar [27, 28]. That is why this well-established culture system was used to identify effect of the fucoidan from brown alga *Fucus evanescens* (FeF) on EGF-induced neoplastic cell transformation.

**Figure 1:**
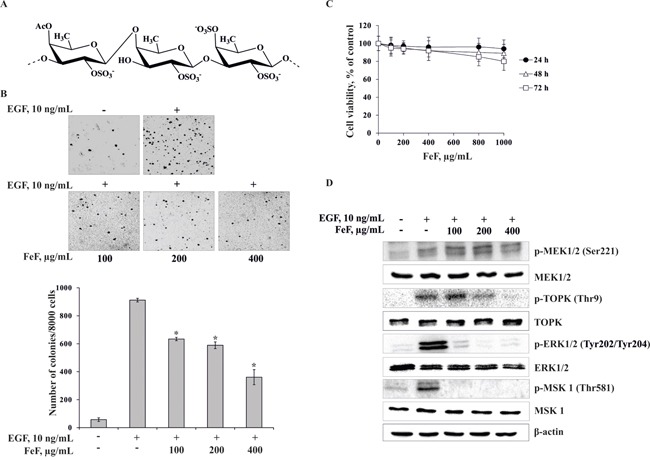
The effect of FeF on EGF-induced neoplastic transformation and molecular mechanism in JB6 Cl41 cells **A.** The chemical structure of FeF. **B.** FeF inhibits EGF-induced anchorage-independent growth of mouse epidermal JB6 Cl41 cells. Data are shown as means ± standard deviation of values from three independent experiments. The asterisk (*) indicates a significant decrease in colony formation in cells treated with FeF compared with the non-treated cells (*p < 0.05). **C.** Cytotoxic effects of FeF on JB6 Cl41 cells. An MTS assay was used after treatment of cells with FeF for 24, 48, and 72 h, respectively. All the experiments were performed in triplicate, and the mean absorbance values were calculated. **D.** FeF inhibits TOPK/ERK/MSK 1 signal transduction in JB6 Cl41 cells as determined by Western Blotting with specific antibodies.

FeF (Figure 1A) was shown to inhibit EGF-induced neoplastic transformation of JB6 Cl41 cells in dose-dependent manner. FeF at concentrations 100, 200, 400 μg/mL decreased the number of transformed cells on 30, 35, 60%, respectively (Figure 1B). It should be noted that the chemopreventive effect of FeF was not due to its cytotoxicity, because it did not possess cytotoxicity at concentration range up to 1 mg/mL even in 3 days of treatment (Figure 1C).

To elucidate molecular mechanism of chemopreventive effect of FeF we tested MEK1/2/TOPK/ERK1/2/MSK 1 pathway, which is link extracellular signals to the machinery that controls fundamental cellular processes such as growth, proliferation, differentiation, migration and apoptosis [29, 30]. Therefore, herein we investigated the effect of FeF on the phosphorylation of MEK1/2, TOPK, ERK1/2, MSK 1 kinases in JB6 Cl41 cells. The FeF was found to inhibit EGF-induced phosphorylation of TOPK, ERK1/2, and MSK 1, but not MEK1/2 kinase. FeF did not influence expression of MEK1/2, TOPK, ERK1/2, and MSK 1 total protein level (Figure 1D).

### The fucoidan inhibits colony formation of colon cancer cells

**Figure 2:**
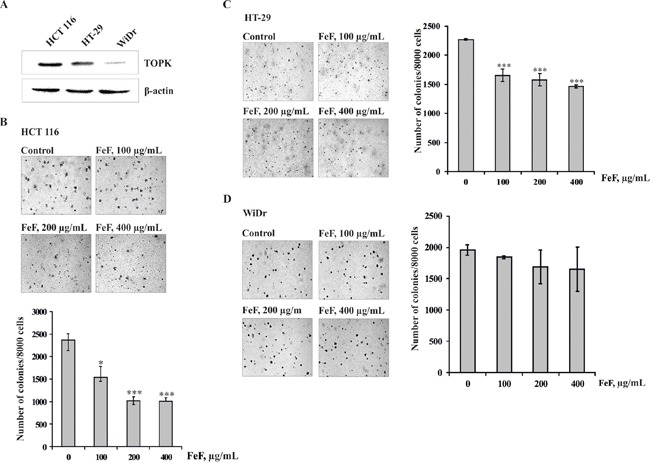
The effects of FeF on colony formation of colon carcinoma cells **A.** Expression of TOPK in colon carcinoma cell lines HCT 116, HT-29 or WiDr. **B, C, D.** The effect of FeF on colony formation in HCT 116, HT-29 or WiDr cells with different level of TOPK expression. Data are shown as means ± standard deviation of values from three independent experiments. The asterisk (*) indicates a significant decrease in colony formation in cells treated with FeF compared with the non-treated cells (*p < 0.05, ***p < 0.001).

Previous studies suggested that TOPK is highly activated in human colon cancer [20]. Therefore, in our study to determine the effect of FeF on colony formation we used colon cancer cell lines HCT 116, HT-29, and WiDr with high, middle, and low expression level of TOPK, respectively (Figure 2A). Cells were maintained with different concentrations of FeF (0 – 400 μg/mL) and colony numbers were counted after culturing for 14 days. The results showed that the fucoidan at 100, 200, and 400 μg/mL inhibited colony formation of HCT 116 on 35, 57, and 58%; HT-29 cells on 21, 31, and 36% and WiDr on 6, 14, and 16%, respectively, compared with non-treated cells (Figure 2B–2D). Overall, our results suggest that inhibitory effect of FeF on colony formation was significant in HCT 116 cells with high expression level of TOPK.

**Figure 3:**
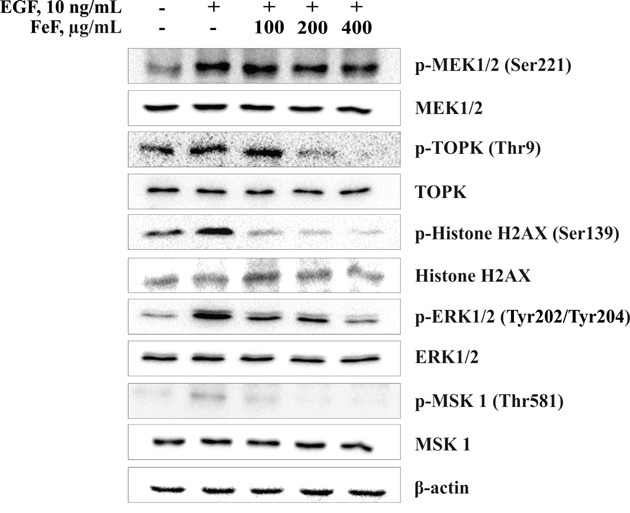
The effect of FeF on EGF-induced phosphorylation of MEK1/2/TOPK/ERK1/2/MSK 1 signaling pathway in HCT 116 cells FeF inhibited EGF-induced phosphorylation of TOPK, H2AX, ERK1/2 or MSK 1, but not MEK1/2 in HCT 116 cells as determined by Western Blotting with specific antibodies.

In addition, to examine the mechanism explaining the chemopreventive effect of FeF on colony formation of colon cancer cells, Western Blotting was used to evaluate the effect of FeF on phosphorylation of MEK1/2, TOPK and its downstream targets, including the phosphorylation of histone H2AX, ERK1/2, and MSK 1 proteins in HCT 116 cells, which were relatively more sensitive to FeF. As expected, phosphorylation of TOPK and down-regulated kinases was inhibited dose-dependently, but not MEK1/2 (Figure 3). It should be noted that FeF did not influence expression of MEK1/2, TOPK, ERK1/2, and MSK 1 total protein level.

### The fucoidan inhibits TOPK kinase activity *in vitro*

**Figure 4:**
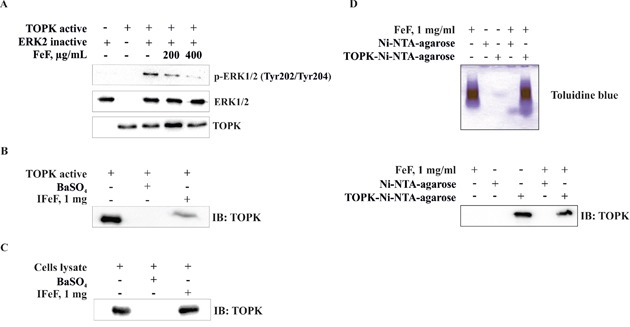
The effect of FeF on TOPK kinase activity *in vitro* and direct binding with TOPK *in vitro* and *ex vivo* **A.** FeF inhibited TOPK kinase activity as determined by Western Blotting. Insoluble fucoidan (IFeF) and TOPK-Ni-NTA-agarose beads were used for binding and pull-down assays as described in Materials and Methods section. **B, C.** Lane 1 is input control (TOPK protein and HCT 116 cell lysate); lane 2 is the negative control, indicating no binding between TOPK and BaSO_4_, and lane 3 shows that TOPK binds with IFeF pellet. **D.** Lane 1 is input control (FeF); lane 2–4 is the negative control, indicating no binding between FeF and Ni-NTA-agarose beads, and lane 5 indicates that FeF binds with TOPK-Ni-NTA-agarose beads.

We next investigated whether the fucoidan might influence kinase activity of TOPK for the inhibition of cells transformation. Data from *in vitro* kinase assays indicated that FeF decreased TOPK kinase activity (Figure 4A). FeF at concentrations of 200 or 400 μg/mL inhibited TOPK kinase activity on 28% or 64%, respectively.

### The fucoidan specifically binds with TOPK *in vitro* and *ex vivo*

The results above showed that the inhibition of EGF-induced neoplastic cell transformation and colony formation of colon cancer cells by FeF involves the suppression of TOPK kinase activity and, subsequently, TOPK downstream signaling pathways. To determine whether FeF exerts its effects by directly interacting with TOPK, we performed pull-down assay using insoluble fucoidan (IFeF) and TOPK-Ni-NTA-agarose beads. We found that TOPK associated with IFeF *in vitro* (Figure 4B, lane 3) and *ex vivo* (Figure 4C, lane 3), but not with BaSO_4_ beads alone (Figure 4B, 4C, lane 2). Moreover FeF was proved to bind with TOPK-Ni-NTA-agarose beads (Figure 4D, lane 5), but not to Ni-NTA-agarose alone by colorimetric method with toluidine blue (Figure 4D, lane 4).

### The fucoidan inhibits growth of colon cancer cells in a xenograft model

**Figure 5:**
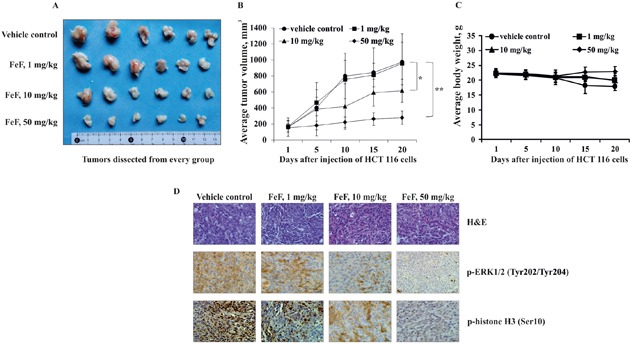
The effect of FeF on tumor growth and phosphorylation of TOPK downstream signaling targets in HCT 116 xenograft mouse model **A.** Tumors dissected from every group. **B.** FeF significantly inhibited colon tumor growth. The average tumor volume of vehicle-treated control mice and FeF-treated mice plotted over 20 days after HCT 116 cells injection. Data are shown as means ± standard deviation of measurements. The asterisk (*) indicates a significant inhibition of tumor growth by FeF (*p < 0.05, **p < 0.01). **C.** FeF does not affect mice body weight. Body weight from treated or untreated groups of mice was measured every other day. **D.** H&E staining and the immunohistochemical analysis of tumor tissues. Treated or untreated groups of mice were euthanized and tumors extracted. Colon cancer tissue slides were prepared with paraffin sections after fixation with formalin and then stained with H&E or p-ERK1/2 and p-histone H3. The magnification of representative photos for H&E and the immunohistochemical staining is ×40.

In order to determine the antitumor activity of FeF *ex vivo*, HCT 116 colon cancer cells were innoculated into the right flank of individual athymic nude mice. Then mice were administered vehicle or FeF (1, 10 or 50 mg/kg of body weight) by oral injection, 3 times a week for 20 days. The treatment of mice with 1, 10, and 50 mg/kg of body weight of FeF was found to inhibit HCT 116 tumor growth by 2, 37, and 72%, respectively, compared with vehicle-treated group (Figure 5A, 5B). It was also shown that mice treated with FeF did not lose body weight compared with vehicle-treated group, which indicated that the dose of FeF used for the experiment had no toxicity to the mice (Figure 5C).

To check whether the antitumor effect of FeF was associated with its inhibition of TOPK activity, tumor tissues from each group were prepared after 20 days of treatment and analyzed for phosphorylation of TOPK downstream targets (p-ERK1/2 (Tyr202/Tyr204) and p-histone H3 (Ser10)) by H&E and immunohistochemistry analyses. The results revealed that expression of p-ERK1/2 and p-histone H3 was decreased after treatment with FeF at either 10 or 50 mg/kg of body weight compared with the control group (Figure 5D). Our data confirm that the fucoidan from brown alga *F. evanescens* suppresses tumor growth by inhibiting TOPK activity *ex vivo*.

## DISCUSSION

The fucoidans, sulfated polysaccharides, are constituents of brown algae and some marine invertebrates (such as sea urchins and sea cucumbers) [31, 32]. The fucoidans isolated from different species of brown algae have extremely diverse chemical structure. And biological activity of the fucoidans was proved to depend on their structural characteristics [33, 34]. The fucoidan from brown alga *Fucus evanescens* (FeF), studied in this work consists of alternating (1→3)- and (1→4)-linked α-L-fucopyranose residues, sulfated and partially acetylated at C2 and/or C4 positions [35, 36]. For the past decade the fucoidans isolated from different species of brown algae have been extensively studied due to their varied biological activities including anticoagulant, antivirus, antitumor, immunomodulatory, anti-inflammatory, antioxidant, and anticomplementary activities [3, 4]. *In vitro*, several mechanisms have been postulated to underlie the anticancer activity of the fucoidans including induction of apoptosis in different types of cancer cells and prevention of angiogenesis and invasion in fibrosarcoma cells [9–18]. However the molecular mechanism of cancer preventive effect of the fucoidans and, moreover, their direct molecular targets has not been identified *in vitro* or *ex vivo*.

The MAP kinases comprise a family of proteins that mediate a succession of discrete signaling cascades. These signaling cascades are activated by a multitude of extracellular stimuli, including a variety of tumor promoters; and they participate in the regulation of a host of cellular functions such as proliferation, cell movement, differentiation, senescence, and death [19]. T-LAK cell-originated protein kinase (TOPK) is a novel serine-threonine kinase that is a member of MAPKK family and is involved in many cellular functions, including tumor development, cell growth, apoptosis, and inflammation [20, 37–39]. Thus, TOPK is found to be a potential target for development of anticancer agents. Our results showed that FeF at nontoxic doses effectively suppressed EGF-induced transformation of JB6 Cl41 cells that was accompanied by decreased phosphorylation of TOPK (Thr9), ERK1/2 (Tyr202/204) and MSK 1 (Thr581), but not MEK1/2 (Ser221), which suggested that FeF attenuated EGF-induced cell transformation by inhibiting of TOPK activity. TOPK/ERK1/2/MSK 1 pathway is likely to play an important role in the cancer preventive activity of FeF. To the best of our (admittedly limited) knowledge, the effect of algal fucoidans on neoplastic cell transformation was not investigated in detail with the notable exception of our works [40, 41], in which the fucoidans were effective in inhibiting EGF-induced cell transformation of JB6 Cl41 cells. The inhibition was found to be associated with the inhibitory effects of polysaccharides on AP-1 activity. However the antitumor activity is known to depend on the structure of the fucoidans, therefore we can affirm that FeF has a unique molecular mechanism of cancer preventive action, which is necessary to be elucidated.

Because TOPK is highly activated in colon cancer cells we checked the idea whether FeF could suppress colony formation of colon cancer cells. In our study we compare the effect of FeF on anchorage-independent growth of colon cancer cells with different level of TOPK expression. It was shown that the fucoidan effectively suppressed colony formation of colon cancer cells HCT 116 with high expression level of TOPK and had less effect on HT-29 and WiDr cells with middle and low TOPK expression level, respectively. The fucoidan was confirmed to inhibit activity of TOPK, The fucoidan was confirmed to inhibit phosphorylation of TOPK (Thr 9), and suggesting fucoidan may inhibit upstream kinase of TOPK, such as Erk-2 (Figure 1D and Figure 3). Further study is needed to explore the effect of fucoidan on feedback loop between TOPK and Erk2 or other upstream kinases of TOPK.

We first determined that FeF could potently suppress TOPK kinase activity *in vitro*. We further conducted *in vitro* and *ex vivo* pull-down assays and confirmed the direct binding between FeF and the TOPK kinase. Recently it was shown that TOPK inhibitor HI-TOPK-032 strongly suppressed TOPK kinase activity and inhibited anchorage-dependent and -independent colon cancer cell growth by reducing ERK-RSK phosphorylation as well as increasing colon cancer cell apoptosis through regulation of the abundance of p53, cleaved caspase-7, and cleaved PARP [42]. However in our study the inhibitory effect of natural compound from brown alga on TOPK was demonstrated for the first time.

Importantly, it was shown that FeF inhibited growth of HCT 116 colon tumor in xenograft animal model. The chemopreventive effect of FeF seemed to be associated with inhibition of the phosphorylation of ERK1/2 and histone H3, direct downstream signaling targets of TOPK. The obtained data strongly indicate that the chemopreventive effect of the fucoidan from brown alga *F. evanescens in vitro* corresponds closely with the results *ex vivo*.

In summary, we provided evidence showing that natural sulfated polysaccharide from brown alga *Fucus evanescens* effectively suppresses EGF-induced transformation of mouse epidermal cells JB6 Cl41 and colony formation of colon cancer cells through TOPK/ERK1/2/MSK 1 signal pathway by inhibiting TOPK kinase activity *in vitro* and *ex vivo*. Overall, our findings support the cancer preventive efficacy of the fucoidan through its targeting of TOPK for the treatment of colon carcinomas.

## MATERIALS AND METHODS

### Reagents and antibodies

The fucoidan FeF was isolated and purified from brown alga *Fucus evanescens* by the modified method [43]. Its structural characteristics were determined as previously described [35, 36].

MTS [3-(4,5-dimethylthiazol-2-yl)-5-(3-carboxymethoxyphenyl)-2-(4-sulfophenyl)-2H-tetrazolium, inner salt] assay kit was purchased from “Promega” (Madison, WI, USA).

Antibodies against p-MEK1/2 (Ser221), p-ERK1/2 (Tyr202/Tyr204), p-TOPK (Thr9), p-histone H2AX (Ser139), p-histone H3 (Ser10), p-MSK 1 (Thr581), MEK1/2, ERK1/2, TOPK, histone H2AX, MSK 1 were obtained from “Cell Signaling Technology” (Massachusetts, USA), β-actin and horseradish peroxidase (HRP) conjugated secondary antibody from rabbit and mouse were purchased from “Santa Cruz” (Dallas, Texas, USA) and “Protein Tech Group” (Chicago, IL, USA), respectively.

The chemiluminescence's detection kit ECL Plus was from “GE Healthcare” (Pittsburgh, PA, USA).

The Basal Medium Eagle (BME), Dulbecco's Modified Eagle medium (DMEM), Minimum Essential medium (MEM), McCoy's 5A Modified medium (McCoy's 5A), Roswell Park Memorial Institute medium (RPMI 1640), phosphate buffered saline (PBS), L-glutamine, gentamicin solution, trypsin, fetal bovine serum (FBS), sodium hydrocarbonate (NaHCO_3_), and agar were purchased from “Sigma” (St. Louis MO, USA) and “Gibco” (Grand Island, NY, USA). All other common chemicals, solvents and reagents were of highest grade available from various commercial sources.

### Cell lines and culture conditions

Mouse epidermal JB6 Cl41 cells (ATCC # CRL-2010) and human colon cancer cells HCT 116 (ATCC # CCL-247™), HT-29 (ATCC # HTB-38), WiDr (ATCC # CCL-218™) were obtained from the American Type Culture Collection (Manassas, VA, USA).

Mouse epidermal JB6 Cl41, human colon cancer HCT 116, HT-29, and WiDr cells were cultured in MEM/5% FBS, DMEM/10% FBS, McCoy's 5 A/10% FBS, and RPMI-1640/10% FBS media, respectively. The cell cultures were maintained at 37°C in humidified atmosphere containing 5% CO_2_.

### MTS assay

To estimate cell viability, JB6 Cl41, HCT 116, HT-29 or WiDr cells (8 × 10^3^/well) were seeded in 96-well plates for 24 h at 37°C in 5% CO_2_ incubator. The attached cells were fed with fresh medium containing various concentrations of the fucoidan from brown alga *F. evanescens* (FeF) (0-1000 μg/mL) for additional 24, 48, 72 h. After culturing for various times, the cytotoxicity of FeF was measured using an MTS assay kit according to the manufacturer's instructions. All the experiments were performed in triplicate, and the mean absorbance values were calculated. The results are expressed as the percentage of inhibition that produced a reduction in absorbance by the fucoidan's treatment compared to the non-treated cells (control).

### Anchorage-independent growth assay (soft agar assay)

JB6 Cl41 cells (2.4 × 10^4^) were exposed to EGF (10 ng/mL) and treated with FeF (0 – 400 μg/mL) in 1 mL of 0.3% Basal Medium Eagle (BME) agar containing 10% FBS, 2 mM L-glutamine, and 25 μg/mL gentamicin. The cultures were maintained at 37°C, in a 5% CO_2_ incubator for 14 days, and the cell's colonies were scored using a microscope «Motic AE 20» (China) and the Motic Image Plus computer program.

To estimate the effect of FeF on colony formation, HCT 116, HT-29 or WiDR (2.4 × 10^4^) were treated with FeF (0 – 400 μg/mL) in 1 mL of 0.3% Basal Medium Eagle (BME) agar containing 10% FBS, 2 mM L-glutamine, and 25 μg/mL gentamicin. The cultures were maintained in a 37°C, 5% CO_2_ incubator for 14 days, and the cell colonies were scored as described above.

### Western blotting

After cells (6 × 10^5^) were cultured in a 10-cm dish overnight, they were treated with FeF (0-400 μg/mL) for 48 h. Then the cells were starved in serum-free medium for another 12 h and treated with EGF (10 ng/mL) for 15 min. The harvested cells were lysed with lysis buffer (50 mM Tris (pH 7.4), 150 mM NaCl, 1 mM EDTA, 1 mM EGTA, 10 mg/mL aprotinin, 10 mg/mL leupeptin, 5 mM phenylmethanesulfonyluoride (PMSF), 1 mM dithiolthreitol (DTT) containing 1% Triton X-100). Insoluble debris was removed by centrifugation at 12000 rpm for 15 min, and protein's content was determined using Bradford reagent “Bio-Rad” (Hercules, California, USA). Lysate protein (20-40 μg) was subjected to 10% sodium-dodecyl sulfate-polyacrylamide gel (SDS PAG) and electrophoretically transferred to polyvinylidene difluoride membranes (PVDF) “Millipore” (Billerica, MA, USA). The membranes were blocked with 5% non-fat milk for 1 h and then incubated with the respective specific primary antibody at 4°C overnight. Protein bands were visualized using an enhanced chemiluminescence reagent (ECL Plus) after hybridization with a HRP conjugated secondary antibody. Band density was quantified using the ImageJ software program (NIH).

### *In vitro* kinase assay

Inactive ERK2 proteins (1 μg) were used as the substrate for an *in vitro* kinase assay with 1.5 μg of active TOPK. Firstly, active TOPK was incubated with FeF (200 and 400 μg/mL) in 1× kinase buffer (25 mM Tris (pH 7.5), 5 mM b-glycerophosphate, 2 mM dithiothreitol (DTT), 0.1 mM Na_3_VO_4_, 10 mM MgCl_2_, and 5 mM MnCl_2_) at 32°C for 15 min. Then inactive ERK2 and 100 mM ATP were added to reaction and incubated at 32°C for 1.5 h. Reactions were stopped by adding 5× SDS sample buffer and then were analyzed by Western blotting. Band density was determined by GS-800 Calibrated Densitometer and quantified using “Quantity One 1-D analysis” software (“Bio Rad”, USA). The percentage of inhibition of kinase activity by FeF was calculated compared with control (sample with TOPK active and ERK2 inactive).

### Preparation of water-insoluble fucoidan (IFeF)

FeF (1 mL, 10%, w/v) was mixed with BaCl_2_ (1 mL, 10%, w/v) and incubated overnight at 4°C. The pellet was washed three times with 96% ethanol. The precipitated insoluble fucoidan (IFeF) was dried at room temperature [41].

### *In vitro* and *ex vivo* IFeF pull-down assays

Human TOPK (1 μg) was incubated with IFeF pellet (1 mg) (or BaSO_4_ alone as a control) in 1× reaction buffer (50 mM Tris (pH 7.5), 5 mM EDTA, 150 mM NaCl, 1 mM DTT, 0,01% NP40, 2 mg/mL bovine serum albumin, 0.02 mM phenylmethylsulfonyl fluoride, 1× protease inhibitors) with gentle rocking overnight at 4°C. The samples were washed five times with washing buffer (50 mM Tris (pH 7.5), 5 mM EDTA, 150 mM NaCl, 1 mM DTT, 0.01% NP40, 0.02 mM phenylmethylsulfonyl fluoride) and TOPK bound to IFeF was analyzed by Western Blotting with the primary antibody against human TOPK. For the *ex vivo* pull-down assay, HCT 116 cell lysate (500 μg) were incubated with IFeF (1 mg) (or BaSO_4_ alone as a control) in 1× reaction buffer. After incubation with gentle rocking overnight at 4°C, the pellet was washed 5 times with washing buffer and proteins bound to IFeF was analyzed by Western Blotting as previously described.

### Bacterial expression and purification of pET46-TOPK (His-TOPK)

The *topk* gene was amplified by PCR and then cloned into pET-46 using a pET-46 Ek/LIC kit (Novagen, USA). His-TOPK fusion protein was expressed in BL21 (DE3) bacteria (Novagen, USA). Bacteria was grown at 37°C to an absorbance of 0.9 - 1.0 at 600 nm and induced with 0.5 mM IPTG overnight at 15°C and harvested by centrifugation. Cell pellets were suspended in 50 mM Tris (pH 8.0) lysis buffer containing 200 mM NaCl and 10 mM imidazole. After sonication and centrifugation, the supernatant fraction was incubated with Ni-NTA-agarose beads “Qiagen” (Germany) overnight at 4°C. Beads were washed with lysis buffer and PBS and used for binding assay as 50% slurry.

### *In vitro* pull-down assay

FeF (1 mg/mL) was incubated with TOPK-Ni-NTA-agarose beads (or Ni-NTA-agarose beads alone as a control; 200 μL, 50% slurry) in the reaction buffer (50 mM Tris (pH 7.5), 5 mM EDTA, 150 mM NaCl, 1 mM DTT, 0.01% Nonidet P-40, 2 mg/mL bovine serum albumin, 0.02 mM phenylmethylsulfonyl fluoride, and 1 mg/mL protease inhibitor mixture). After incubation with gentle rocking overnight at 4°C, the beads were washed 5 times with washing buffer (50 mM Tris (pH 7.5), 5 mM EDTA, 150 mM NaCl, 1 mM DTT, 0.01% Nonidet P-40, 2 mg/mL bovine serum albumin, 0.02 mM phenylmethylsulfonyl fluoride).

The binding between the TOPK-Ni-NTA-agarose beads and FeF was examined by color reaction with toluidine blue stain. Firstly, FeF (control) and TOPK-Ni-NTA-agarose-FeF complex were applied to the agarose gel electrophoresis. The electrophoresis was carried out in Tris-borate buffer pH 8.3 (0.09 M Tris, 0.4 M H_3_BO_3_, 0.01 M EDTA) at 25 mA for 2h. The gel was stained with 0.025 % solution of toluidine blue in acetic acid. Band density was determined by GS-800 Calibrated Densitometer and quantified using “Quantity One 1-D analysis” software “Bio-Rad” (Hercules, California, USA).

### Xenograft mouse model

Athymic nude mice [6- to 9-week old] were obtained from Beijing HFK Bioscience CO., LTD (Beijing, China) and maintained under ‘specific pathogen-free’ conditions based on the guidelines established by the Laboratory of Animal Center of the Fourth Military Medical University. Mice were divided into different groups (*n* = 6 of each group). HCT 116 colon cancer cells (3 × 10^6^/0.1 ml 1× PBS) were injected subcutaneously into the right flank of each mouse. FeF (1, 10, and 50 mg/kg of body weight) or vehicle was administered by oral injection three times a week for 20 days. Tumor volumes and body weights were measured every other day. The volume (V) of the tumor was calculated using the formula: volume (v) = 0.52 (length (l) × width (w) × height (h)). Treated or non-treated groups of mice were euthanized and tumors extracted. Colon cancer tissue slides were prepared with paraffin sections after fixation with formalin and then stained with hematoxylin and eosin (H&E) staining or p-ERK1/2 (Tyr202/Tyr204) and p-histone H3 (Ser10).

### Statistical analysis

All assays were performed at least in triplicate. The results are expressed as the mean ± standard deviation (SD). A Student's t-test was used to evaluate the data with the following significance levels: *p < 0.05, **p < 0.01, ***p < 0.001.
